# 多材料3D打印技术制作用于毛细管电泳的非接触电导/激光诱导荧光二合一检测池

**DOI:** 10.3724/SP.J.1123.2021.02021

**Published:** 2021-08-08

**Authors:** Piwang ZHANG, Liye YANG, Qiang LIU, Shangui LU, Ying LIANG, Min ZHANG

**Affiliations:** 1.桂林电子科技大学生命与环境科学学院, 广西 桂林 541004; 1. School of Life and Environmental Sciences, Guilin University of Electronic Technology, Guilin 541004, China; 2.品创检测(广西)有限公司, 广西 桂林 541004; 2. Pinchuang Testing (Guangxi) Corporation, Guilin 541004, China

**Keywords:** 毛细管柱上检测, 组合检测, 电容耦合非接触电导检测, 激光诱导荧光, 毛细管电泳, on-capillary detection, dual detection, capacitively coupled contactless conductivity detection (C^4^D), laser induced fluorescence (LIF), capillary electrophoresis (CE)

## Abstract

利用多材料3D打印技术研制了用于毛细管电泳(CE)的二合一检测池,实现了电容耦合非接触电导(C^4^D)与共聚焦激光诱导荧光(LIF)两种检测方法在毛细管柱上同一位置同时检测。3D打印的检测池采用了导电的复合聚乳酸(PLA)材料制作C^4^D的屏蔽层,采用普通的绝缘PLA材料支撑C^4^D金属管电极并隔离屏蔽层。两根金属管电极通过“打印-暂停-打印”的方式嵌入到检测池中,两电极被2 mm厚的导电屏蔽层隔开,在屏蔽层中有一直径为1 mm的圆形通孔用于LIF检测。该检测池与带流通式进样接口的自组装CE系统联用,用于同时检测无机离子和异硫氰酸荧光素(FITC)标记的氨基酸。研究优化了C^4^D激励信号频率与电泳缓冲液浓度,选用的电泳缓冲溶液为10 mmol/L 3-吗啉丙烷-1-磺酸(MOPS)与10 mmol/L二(2-羟乙基)亚氨基三(羟甲基)甲烷(Bis-Tris)的混合溶液,选用C^4^D激励频率为77 kHz。二合一检测池应用于内径为25 μm的毛细管时,C ^4^D对Na^+^、K^+^和Li^+^的检出限分别为2.2、2.0和2.6 μmol/L; LIF对荧光素和FITC的检出限分别为7.6和1.7 nmol/L。两种检测方法的相对标准偏差在0.3%至4.5%之间(*n*=3),工作曲线的相关系数*r* ^2^≥0.9904。采用3D打印技术可以在实验室内实现复杂结构的制作,降低了制作的成本,且便于方法的推广和改进。

## Abstract (en)

Dual detection, which simultaneously employs two complementary detection methods, is a useful approach to enhance the selectivity and sensitivity of capillary electrophoresis (CE). Through dual detection, multiple classes of analytes with different structural and chemical characteristics can be sensitively detected using a single CE method. In addition, the comigrating peaks can be distinguished by comparing the signal outputs of two detectors with different selectivities. Typically, dual detection is achieved by coupling two detectors in series along a capillary. However, in this approach, it is inconvenient to evaluate the signal outputs of the two detectors. The two detectors present differences in their corresponding effective capillary lengths and dead volumes of the detection cell. Therefore, detectors that combine two or three detection methods in a single detection point are proposed to address this issue.

In this work, to fabricate a combined detector in a simple and low-cost manner, multimaterial 3D printing technology is employed. A two-in-one detection cell that combines capacitively coupled contactless conductivity detection (C^4^D) and confocal laser-induced fluorescence (LIF) detection was fabricated by 3D printing functional materials. In 3D printing, conductive composite polylactic acid (PLA, Proto-pasta) filaments and normal nonconductive PLA filaments were employed. The conductive material was used to build a C^4^D shielding layer that was electrically grounded. The nonconductive PLA was used as an electrical insulator placed between the shielding layer and C^4^D electrodes, which were two stainless-steel tubes (0.4 mm i.d. and 5 mm length). To embed the electrodes into the nonconductive material, a “print-pause-print” approach was applied. After building two chambers for housing electrodes using nonconductive PLA, the 3D printing was paused, following which the two electrodes were manually installed. Printing was then resumed, and the remaining part was built. The two electrodes were 2 mm apart, and the gap between them was filled with a conductive material for shielding to eliminate stray capacitance. A through-hole (1 mm i.d.) was placed between the middle conductive shielding layer for LIF detection. The size of the detection cell was 60 mm×29 mm×7.2 mm. The cell was screwed onto an XYZ stage to precisely align the light path of LIF detection, which was realized using a TriSep ^TM^-2100LIF detector equipped with a 473 nm laser. C^4^D detection was achieved using a TraceDec detector equipped with a ChipCE adaptor. The two-in-one detector was coupled with a lab-made CE system that had a flow-through injection interface.

Use of the detection cell allows the simultaneous detection of inorganic cations and fluorescein isothiocyanate (FITC)-labeled amino acids. The C^4^D excitation frequency and buffer concentration were then optimized. A mixture of 10 mmol/L 3-(*N*-morpholino)propanesulfonic acid (MOPS) and 10 mmol/L bis(2-hydroxyethyl)amino-tris(hydroxymethyl)methane (Bis-Tris) was selected as the background electrolyte as a compromise of C^4^D signal-to-noise ratio (*S/N*) and separation efficiencies of amino acids. The C^4^D excitation frequency was set to 77 kHz with *S/N*=233±8 for 200 μmol/L Na ^+^. The baseline separation of Na^+^, K^+^, Li^+^, FITC, fluorescein, histidine (His), lysine (Lys), tryptophan (Trp), phenylalanine (Phe), alanine (Ala), and glycine (Gly) was achieved with a 25 μm i.d.×365 μm o.d.×45 cm (35 cm effective length) capillary and -10 kV separation voltage. The limits of detection (LODs) of C ^4^D for Na^+^, K^+^, and Li^+^were 2.2, 2.0, and 2.6 μmol/L, respectively. The LODs of LIF for fluorescein and FITC were 7.6 and 1.7 nmol/L, respectively. The relative standard deviations (RSDs) of the two detection methods were within the range of 0.3%-4.5% (*n*=3). The *r* ^2^ of the calibration curves was ≥0.9904. Thus, 3D printing technology is a simple and low-cost approach to implement complex designs, including those that are difficult to fabricate by traditional “workshop” technologies.

便携式毛细管电泳(CE)仪器已被用于环境中多种类型污染物的现场快速检测^[1,2]^。CE柱上检测方法常用的有紫外-可见吸光光度法(UV-Vis)^[3,4]^、荧光光度法^[5]^和电容耦合非接触电导法(C^4^D)^[6,7,8]^。各单一检测方法直接检测时适用的目标物范围是有限的,虽然可以通过间接检测的手段拓展适用的目标物范围^[9]^,但是间接检测的基线稳定性较差,不利于低含量目标物的测定。因此有学者将多种检测器串联在一根毛细管上实现了不同类型目标物的先后顺序检测^[10]^。然而,多种检测器串联是多点检测,各检测器对应的有效分离长度不同,导致它们之间的数据校正较为复杂,给数据分析带来不便;此外,对于环境现场快速分离分析而言,串联多个检测器不利于便携式仪器的小型化。

为了解决检测器串联使用的弊端,曾有学者^[11,12,13,14,15]^将多种检测方法组合在同一检测池上,实现毛细管柱上两种^[11,12,13,14]^或3种^[15]^方法单点检测。如将C^4^D和荧光^[13,14]^方法组合,可以对金属离子和氨基酸实现同时分析^[13,14]^。多合一检测器使各检测方法对应的毛细管有效长度一致,因此方便了各方法之间的校正和对比。

随着技术的不断发展,3D打印凭借其快速性、低成本和可定制性等优势受到越来越多的关注^[16,17,18]^。已有文献报道利用3D打印技术研制毛细管柱上UV-Vis检测器^[19]^、发光二极管诱导荧光检测器^[20,21]^等。尚未见3D打印技术制作毛细管柱上C^4^D的文献报道,也未见3D打印制作多合一检测器的报道。

本研究尝试通过多材料3D打印技术研制C^4^D与共聚焦激光诱导荧光(LIF)单点同时检测的组合检测器。研究采用导电的打印材料制作C^4^D屏蔽层,用于阻断两电极间的耦合电容并屏蔽外界信号的干扰;绝缘材料作为管状电极内部的支撑,还起到电极与屏蔽层的隔离作用;通过在中间屏蔽层上打印大小为1 mm的通孔,实现了LIF与C^4^D的组合检测,利用激光光源代替以往组合检测器使用的发光二极管光源,获得了更高的荧光检测灵敏度。

## 1 实验部分

### 1.1 试剂与材料

所有试剂和药品均购自上海阿拉丁生化科技有限公司。实验用水均为电阻率18.2 MΩ·cm的超纯水。背景电解质(BGE)为浓度比1∶1(10 mmol/L)的3-吗啉丙烷-1-磺酸(MOPS)与二(2-羟乙基)亚氨基三(羟甲基)甲烷(Bis-Tris)混合溶液,使用前由0.45 μm滤膜过滤。标准溶液由荧光素钠(Flu)、组氨酸(His)、赖氨酸(Lys)、色氨酸(Trp)、苯丙氨酸(Phe)、丙氨酸(Ala)、甘氨酸(Gly)、氯化钠、氯化钾和氯化锂等溶解在超纯水中配制。异硫氰酸荧光素(FITC)、丙酮、吡啶、四硼酸钠等用于氨基酸的衍生化反应。

熔融石英毛细管(内径25 μm,外径365 μm)购自河北永年锐沣色谱器件有限公司,用火焰去除涂层制作LIF检测窗口。含炭黑的Proto-pasta导电3D打印材料(美国Protoplant)和白色聚乳酸(PLA)材料(深圳纵维立方科技)直径均为1.75 mm。

### 1.2 实验装置

实验采用带流通式进样接口的自组装CE系统(见图1)。氮气钢瓶通过减压阀与微型数字压力控制器(990-005123-050,美国Parker)连接,可产生0~345 kPa(0~50 psi)的恒定压力输出至缓冲溶液瓶(11)。流通式进样装置由KPP微型蠕动泵(上海卡川尔流体科技,12)、六通进样阀(美国VICI, 9)、电磁阀(991-000515-004,美国Pneutronics, 13)、三通接头(10)和接地不锈钢管组成。CE所需高压(-10 kV)由Q101N-5直流高压电源模块(美国EMCO)产生。自组装CE系统的控制由LabVIEW(美国NI)编写的程序结合USB-1208FS-Plus数据采集卡(美国Measurement Computing)、BMZ-06R1-E继电器模块(深圳泽炜电器)实现。进样及电泳分离的详细流程可参考本团队前期发表的文章^[22]^。

**图1:**
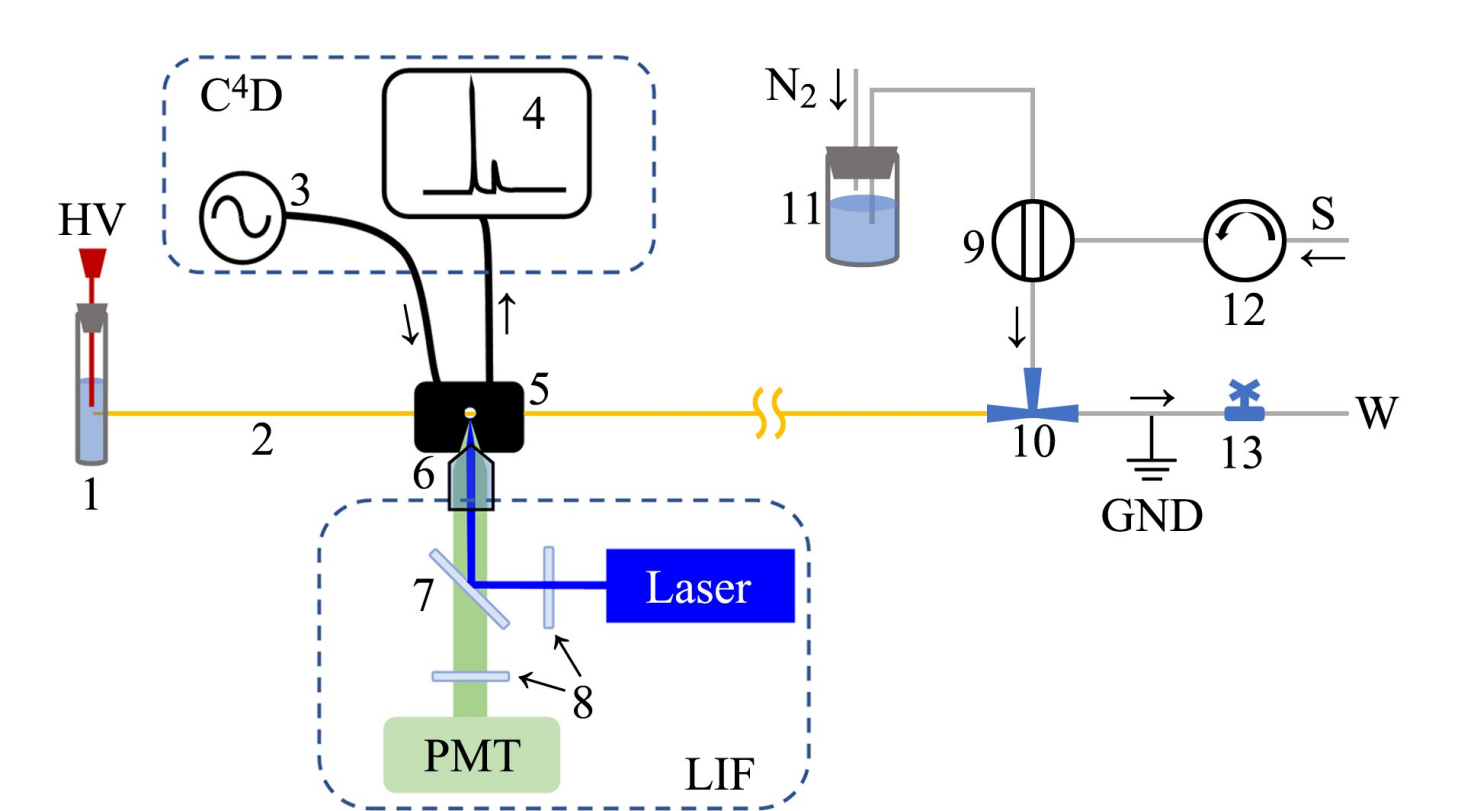
电泳装置的示意图

3D打印检测池的C^4^D电极通过屏蔽线以及BNC接头与配备ChipCE接口的TraceDec检测器(奥地利Innovative Sensor Technologies)连接。LIF检测由TriSep^TM^-2100LIF检测器(上海通微,激光波长473 nm)实现。检测池与XYZ轴手动精密微调平台(山东安赢)通过螺丝固定,实验通过调整微调平台获取最佳LIF检测灵敏度。

### 1.3 检测池的设计及3D打印

实验的计算机辅助设计软件为Solidworks,打印参数设置和模型切片由Slic3r Prusa Edition软件完成。3D打印机为Prusa i3 MK3 MMU2(捷克Prusa Research)。检测池的设计如图2所示,尺寸为60 mm×29 mm×7.2 mm。检测池的屏蔽层(1)由导电材料打印,内绝缘层(2)由白色PLA材料打印。两根管状电极(3)为内径0.4 mm、长5 mm的不锈钢针管,与同轴电线(4)的内导线通过焊接导通。两电极之间的屏蔽层(2 mm厚)留有一个1 mm直径的垂直通孔(5)用于LIF检测。为将电极及导线嵌入检测池,当用于放置电极的槽孔打印成型后,将打印机暂停,手工操作装入;同时,将同轴电线屏蔽层的编织网拆散,平铺在打印层上,再使用加热的电烙铁对平铺的屏蔽线(6)按压,将其嵌入到导电层中以形成稳固的连接。在安装电极和屏蔽线后,使用万用表检测确保屏蔽层与电极不导通,而电极与内导线、屏蔽线与屏蔽层导通,打印机再恢复工作完成剩余部分打印。毛细管(7)去除部分涂层后插入检测池中。检测池通过两个螺孔(8)与精密微调平台固定。

**图2:**
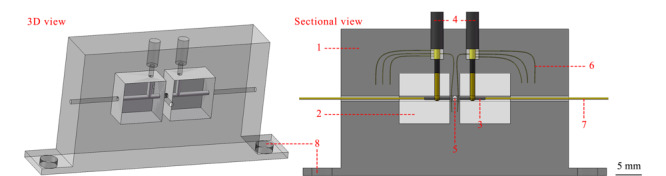
二合一检测池的设计图

## 2 结果与讨论

### 2.1 二合一检测池的设计参数

检测池设计时需要兼顾两种检测方法的灵敏度。实验分别制作了0.5 mm和1 mm直径的通孔用于LIF检测。当使用0.5 mm通孔时,激光光斑被部分阻挡;而通孔直径为1 mm时,光斑能无阻挡地聚焦在毛细管上,因此最终选择通孔直径为1 mm。通孔直径选定为1 mm后,为确保打印的屏蔽层上不存在空隙,提供较好的屏蔽效果,通孔边缘的两层导电材料最薄处分别设计为0.5 mm,因此最终C^4^D电极间距为2 mm。

### 2.2 C^4^D激励频率及电泳缓冲液浓度的优化

实验选用浓度比1∶1的Bis-Tris+MOPS缓冲溶液作为BGE,该溶液能够提供较为平稳的C^4^D检测基线^[22]^,且其pH为6.8,在此条件下大多数常见的阴阳离子都处于解离状态,能够兼顾阴阳离子的同时分离和C^4^D检测。应用C^4^D检测时,激励频率(*f*)的最优值与BGE的电导率值(浓度)相关,当使用较高浓度的BGE时,应施加较高*f*以获得最优的*S/N*^[8]^。通过设置TraceDec检测器,考察了不同浓度BGE检测200 μmol/L Na^+^的信号峰高和*S/N*,结果见图3。图3表明,当BGE浓度为5 mmol/L(5 mmol/L Bis-Tris+5 mmol/L MOPS)时,C^4^D检测器只在38 kHz时有信号响应;当使用10 mmol/L BGE时,检测器在*f*=38、77和157 kHz时都能产生输出信号,在77 kHz获得最优*S/N*=233±8;进一步升高缓冲溶液浓度至20 mmol/L,在*f*=38~307 kHz范围内都能检测到信号,而获得最优*S/N*值494±8对应的*f*为157 kHz。最优*f*随溶液浓度升高而升高的变化趋势与文献^[8]^报道结论相符。

**图3:**
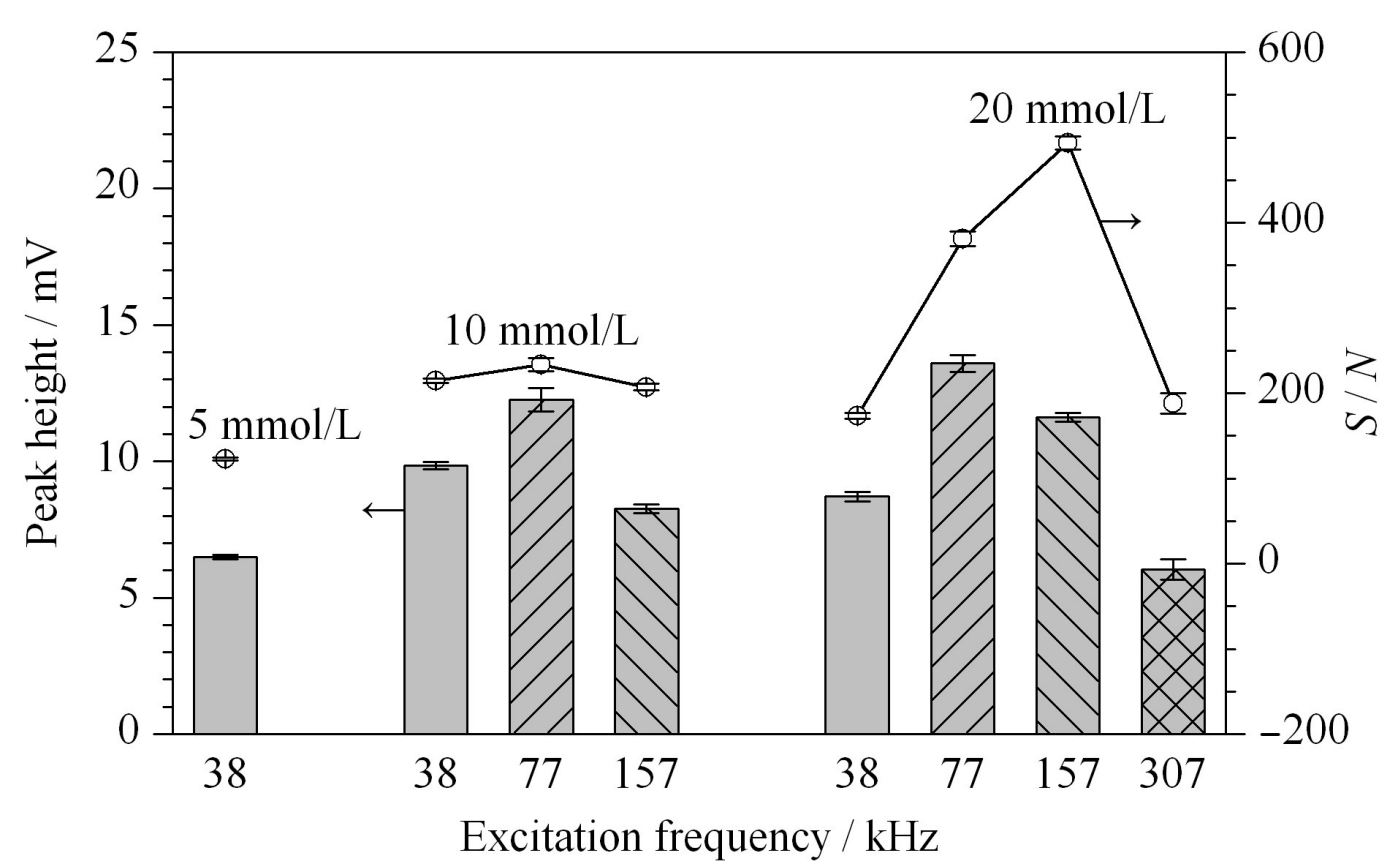
缓冲液浓度和激励频率对C^4^D峰高和信噪比的影响(*n*=3)

但使用20 mmol/L BGE时,电渗流速度下降,导致净迁移速率较小的氨基酸信号峰展宽,因此综合考虑C^4^D检测的*S/N*以及氨基酸的分离柱效,后续实验选用的电泳缓冲溶液浓度为10 mmol/L, *f*=77 kHz。

### 2.3 二合一检测器的分析性能

应用3D打印检测池对含Na^+^、K^+^和Li^+^的离子溶液进行电导检测,对含荧光素和FITC溶液进行荧光检测,考察其分析性能,结果如表1所示。在表1所示的浓度范围内,各目标物浓度与峰面积有良好的线性关系,工作曲线*r*^2^≥0.9904。对于阳离子的C^4^D检测,方法检出限为2.0~2.6 μmol/L,峰面积的相对标准偏差(RSD)为0.3%~1.6%;对于Flu和FITC的LIF检测,方法检出限分别为7.6和1.7 nmol/L,峰面积的RSD为1.6%~4.5%。对比文献^[13,14,15]^报道的组合检测器,虽然本实验采用了更细内径的25 μm毛细管,但3D打印检测池在C^4^D检测上获得了相近的分析性能;在荧光检测上,由于采用了激光光源,所以本实验获得的灵敏度和检出限更优。

**表1:** 二合一检测器的工作曲线范围、检出限和相对标准偏差(*n*=3)

Detector	Ion	Working curve range/(μmol/L)	*r* ^2^	LOD/ (μmol/L)	RSD/%
C^4^D	K^+^	2.0-500	0.9983	2.0	0.8-1.6
	Na^+^	2.2-500	0.9984	2.2	0.4-1.2
	Li^+^	2.6-500	0.9984	2.6	0.3-1.5
LIF	Flu	0.0076-40	0.9904	0.0076	2.1-4.5
	FITC	0.0017-40	0.9947	0.0017	1.6-3.2

Flu: fluorescein; FITC: fluorescein isothiocyanate.

实验采用二合一检测器对K^+^、Na^+^、Li^+^和FITC标记的氨基酸^[13,14]^混合溶液进行电泳分离检测,两种检测方法所得的电泳谱图见图4。图4表明,C^4^D检测器对摩尔电导率较大的阳离子有着较好的响应,且能够清晰的确定电渗流位置,但对于相对分子质量较大、摩尔电导率值较低的氨基酸衍生产物响应较差。LIF对含有荧光基团的化合物能够灵敏检测,但无法确定电渗流位置。对两种检测方法都能响应的氨基酸衍生物的信号峰进行对比分析,发现两种检测方法所得的迁移时间高度一致(相对偏差的绝对值≤0.44%),但是C^4^D所得的半峰宽值较大,为LIF检测所得数值的1.41±0.16倍(*n*=3)。可能的原因是:(1)C^4^D检测池的宽度稍大;(2)TraceDec检测器自带的信号处理算法造成了峰展宽^[23]^。应用二合一检测池可以对浓度差别较大的不同类型目标物实现同时检测,发挥了各检测方法的优势,有助于实现对成分复杂样品的快速检测。

**图4:**
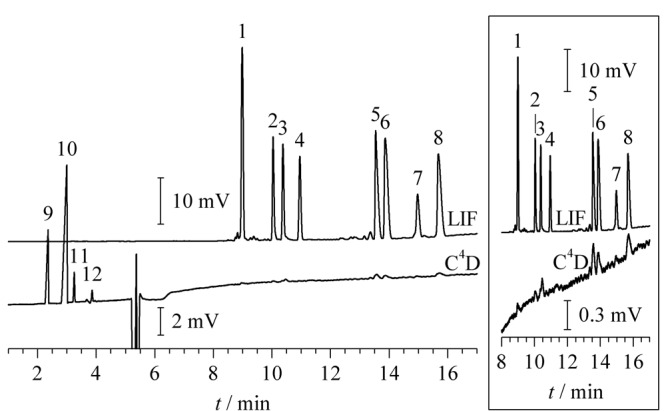
二合一检测器检测的电泳谱图

## 3 结论

本研究采用多材料3D打印技术,将导电打印材料与绝缘材料搭配使用,研制了C^4^D/LIF二合一检测池。3D打印技术实现了复杂结构的制作,并降低了成本,简化了流程,也方便实验室之间进行方法验证。相较于文献报道的组合检测器,本装置有着相近或者更优的检测性能。二合一检测器解决了多检测器串联式检测方法响应不同步、互相之间校正困难的问题;而相较于单一的检测器有更宽的检测范围,具有较好的应用前景。
